# SMARCA4-Deficient Undifferentiated Tumor of the Right Colon in a Patient With Ulcerative Colitis

**DOI:** 10.7759/cureus.113940

**Published:** 2026-08-04

**Authors:** Elizabeth Reynolds, Fergus Reid, Rawan ElManfalouty, Ahmed Holayel, Shailesh Agrawal, Konstantinos Kamposioras

**Affiliations:** 1 Department of Surgery, East Lancashire Hospitals NHS Trust, Blackburn, GBR; 2 Department of Surgery, Stockport NHS Foundation Trust, Stockport, GBR; 3 Department of Medical Oncology, The Christie NHS Foundation Trust, Manchester, GBR; 4 Department of Oncology, University Hospitals of Leicester NHS Trust, Leicester, GBR; 5 Department of Pathology, Stockport NHS Foundation Trust, Stockport, GBR

**Keywords:** colorectal cancer, folfoxiri, gastrointestinal obstruction, subtotal abdominal colectomy, ulcerative colitis

## Abstract

Undifferentiated gastrointestinal tumors with SMARCA4 deficiency are a rare subset of malignancies that often present as advanced, rapidly progressive disease. SMARCA4 loss is identified by immunohistochemistry, and affected tumors demonstrate distinct morphological features. Ulcerative colitis is a chronic inflammatory disorder that predisposes patients to colitis-associated colorectal cancer through a variety of genetic alterations.

We present the case of a 31-year-old patient with a SMARCA4-deficient undifferentiated right-sided colon cancer detected during surveillance colonoscopy for ulcerative colitis. We also review the potential association between ulcerative colitis and SMARCA4-deficient undifferentiated gastrointestinal tumors.

## Introduction

SMARCA4 deficiency results from alterations in the SMARCA4 gene, which encodes a protein involved in chromatin remodeling that is essential for deoxyribonucleic acid (DNA) transcription [1]. Undifferentiated tumors with SMARCA4 deficiency (SMARCA4-deficient undifferentiated tumor (SMARCA4-dUT)) are rare and are often rapidly progressive. They typically present at an advanced stage, frequently with metastatic disease, and are associated with resistance to chemotherapy as well as an overall poor prognosis. Although SMARCA4-deficient tumors most commonly arise in the lung, they may rarely occur in other sites, including the gastrointestinal tract [2]. SMARCA4-dUT of the colon is exceedingly rare, with only one other case reported in the literature [3].

The morphological features of SMARCA4-dUT include a lack of differentiation, abundant eosinophilic cytoplasm, irregularly shaped nuclei, and large, poorly cohesive epithelioid cells. Complete loss of SMARCA4 expression can be demonstrated by immunohistochemistry [4].

Although chemotherapy and immunotherapy have been administered in some cases, the rarity of SMARCA4-dUT has precluded the development of standardized treatment guidelines due to limited available evidence. In a systematic review of treatment responses, gastrointestinal and gynecological SMARCA4-dUTs showed poorer responses to immune checkpoint inhibitors and shorter survival than in thoracic tumors [2].

Ulcerative colitis (UC) is associated with an increased risk of colorectal cancer because chronic inflammation promotes repeated cycles of tissue injury and repair [5]. Established risk factors include the extent and duration of UC, the severity of inflammation, and younger age at diagnosis. The estimated risk of colorectal cancer increases from approximately 2% after 10 years of disease to 18% after 30 years [6]. The incidence of colitis-associated colorectal cancer (CAC) has declined in recent years, possibly because of improved medical therapy that more effectively controls active disease and reduces chronic inflammation [5,6]. The genetic alterations associated with CAC are broadly similar to those observed in sporadic colorectal cancer, with the most common mutation involving TP53, followed by APC, KRAS, and SMAD4 [5,7].

The SMARCA4 gene, also known as BRG1, encodes a key component of the SWI/SNF chromatin-remodeling complex, which plays an important role in suppressing inflammation and preventing tumor formation in intestinal epithelial cells. BRG1 expression is reduced in patients with inflammatory bowel disease [8].

## Case presentation

A 31-year-old woman attended for surveillance colonoscopy following a routine gastroenterology outpatient review. Eight years earlier, she had been diagnosed with moderate pancolonic ulcerative colitis after presenting with bloody stools, abdominal pain, and elevated fecal calprotectin levels. She had previously received several oral therapies before commencing vedolizumab two years earlier following a significant disease flare. Since then, she had remained clinically stable and asymptomatic, with no previous hospital admissions or surgical interventions related to her ulcerative colitis. She had no family history of colorectal cancer, was otherwise fit and well, and her only additional medical history included chronic fatigue and endometriosis, neither of which affected her daily activities.

The colonoscopy was performed as part of routine low-risk surveillance and demonstrated patchy mild-to-moderate pancolitis, together with a suspicious ulcerated lesion at the hepatic flexure. The lesion was biopsied, although tissue acquisition was technically challenging. She was subsequently referred via the two-week suspected cancer pathway. CT of the chest, abdomen, and pelvis demonstrated annular thickening at the hepatic flexure measuring more than 8 cm, with associated bowel wall edema and multiple enlarged adjacent lymph nodes. There was no evidence of distant metastatic disease.

The CT findings could not distinguish between an inflammatory mass and a malignant lesion, and the initial biopsy specimens demonstrated only low- to moderate-grade dysplasia. Following discussion at the colorectal multidisciplinary team (MDT) meeting, a repeat colonoscopy with additional biopsies was recommended because of concern that the lesion represented a more advanced process than indicated by the initial histology. This was performed one month after the initial endoscopy and demonstrated progression of the lesion to an obstructing polypoid tumor. However, the repeat biopsies again showed only moderate dysplasia in the presence of extensive inflammation. The specimens were therefore referred to the regional cancer center for specialist pathological review.

The patient was counseled regarding the high likelihood of an underlying malignancy. Following further MDT discussion and consultation with the patient, it was decided to proceed with a subtotal colectomy and end ileostomy rather than a segmental colectomy in order to remove the tumor and provide definitive surgical management of her ulcerative colitis.

**Figure 1:**
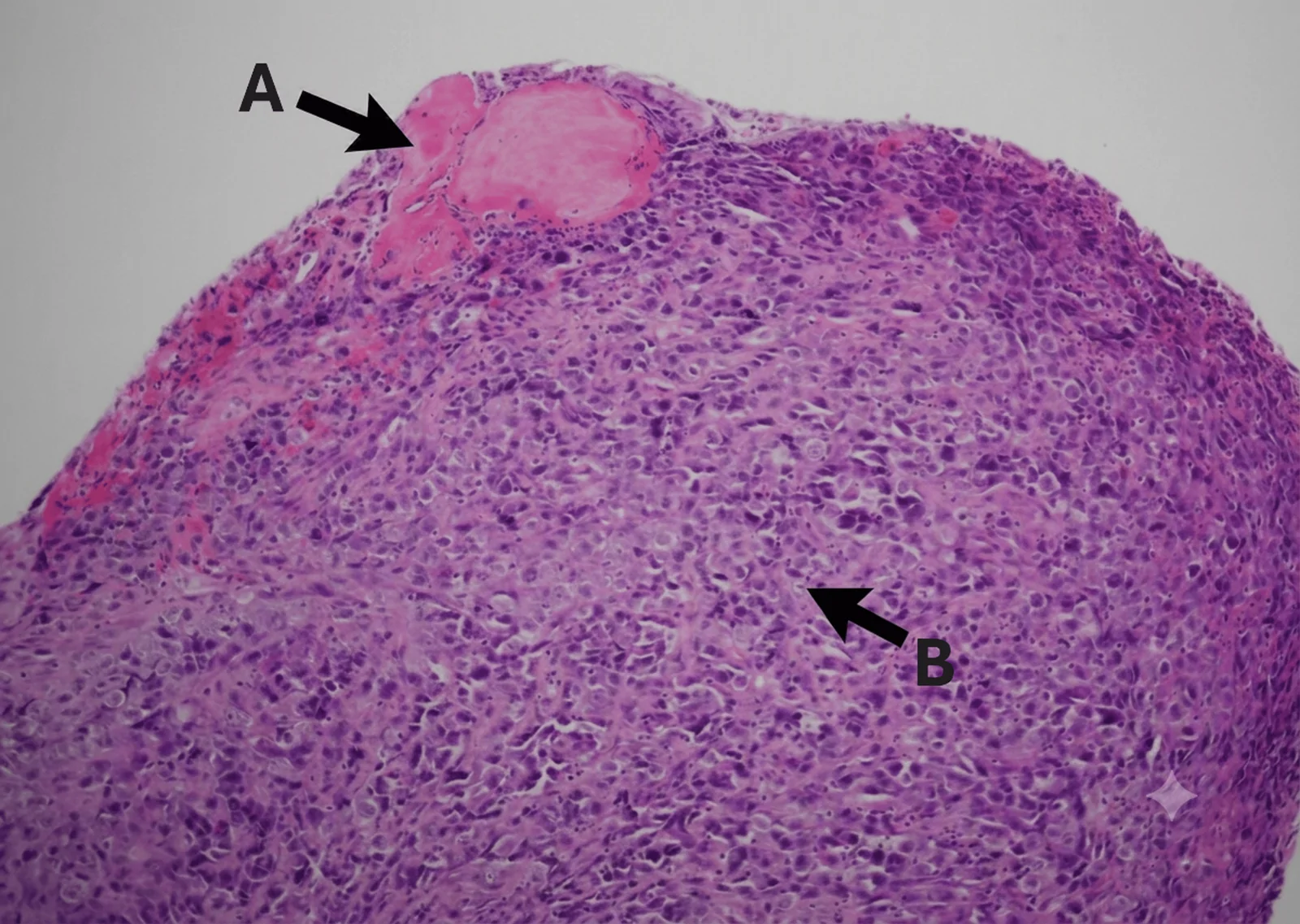
Low-power view of the tumor showing diffuse infiltration by high-grade malignant cells arranged in solid sheets, with pale eosinophilic cytoplasm, pleomorphic vesicular nuclei, and focal rhabdoid morphology. Extensive tumor necrosis is also present. (A) Area of extensive tumor necrosis (characterized by pale, acellular eosinophilic debris). (B) Solid sheet of malignant tumor cells displaying diffusely infiltrating high-grade features, marked pleomorphic vesicular nuclei, and pale eosinophilic cytoplasm, with focal rhabdoid morphology.

One week before surgery, specialist review of both biopsy specimens identified disorganized tumor cells with rhabdoid morphology and pleomorphic vesicular nuclei. Immunohistochemistry demonstrated that the tumor cells were weakly and patchily positive for MNF116 and SATB2 (weak nuclear staining), with focal positivity for AE1/AE3, synaptophysin (weak), and CD138 (weak). The tumor cells were negative for CK7, CK20, CDX2, ERG, CD34, Melan-A, S100, SOX10, CD30, LCA, and INSM1, with retained INI1 expression. Loss of SMARCA4 expression was demonstrated. Overall, the morphological and immunophenotypic findings were consistent with an SMARCA4-deficient undifferentiated carcinoma (SWI/SNF complex-deficient undifferentiated carcinoma of the gastrointestinal tract) (Figure 1). This rare tumor type is thought to arise through dedifferentiation of a lower-grade carcinoma.

The patient was informed of the biopsy results but was admitted one week before surgery because of significant weight loss, diarrhea, and fatigue. Her inflammatory markers were elevated, and repeat CT demonstrated inflammatory changes throughout the colon, with no evidence of tumor-related obstruction or perforation. She was treated with corticosteroids, resulting in clinical improvement, and received a blood transfusion to optimize her condition before undergoing the planned surgery.

Initial laparoscopic assessment revealed a large tumor, prompting early conversion to an open procedure. As planned, a subtotal colectomy with end ileostomy was performed. Although there was regional lymphadenopathy, there was no gross evidence of peritoneal disease or distant metastases, and the tumor was not adherent to adjacent structures. The patient made an uneventful postoperative recovery and was discharged without complications.

**Figure 2:**
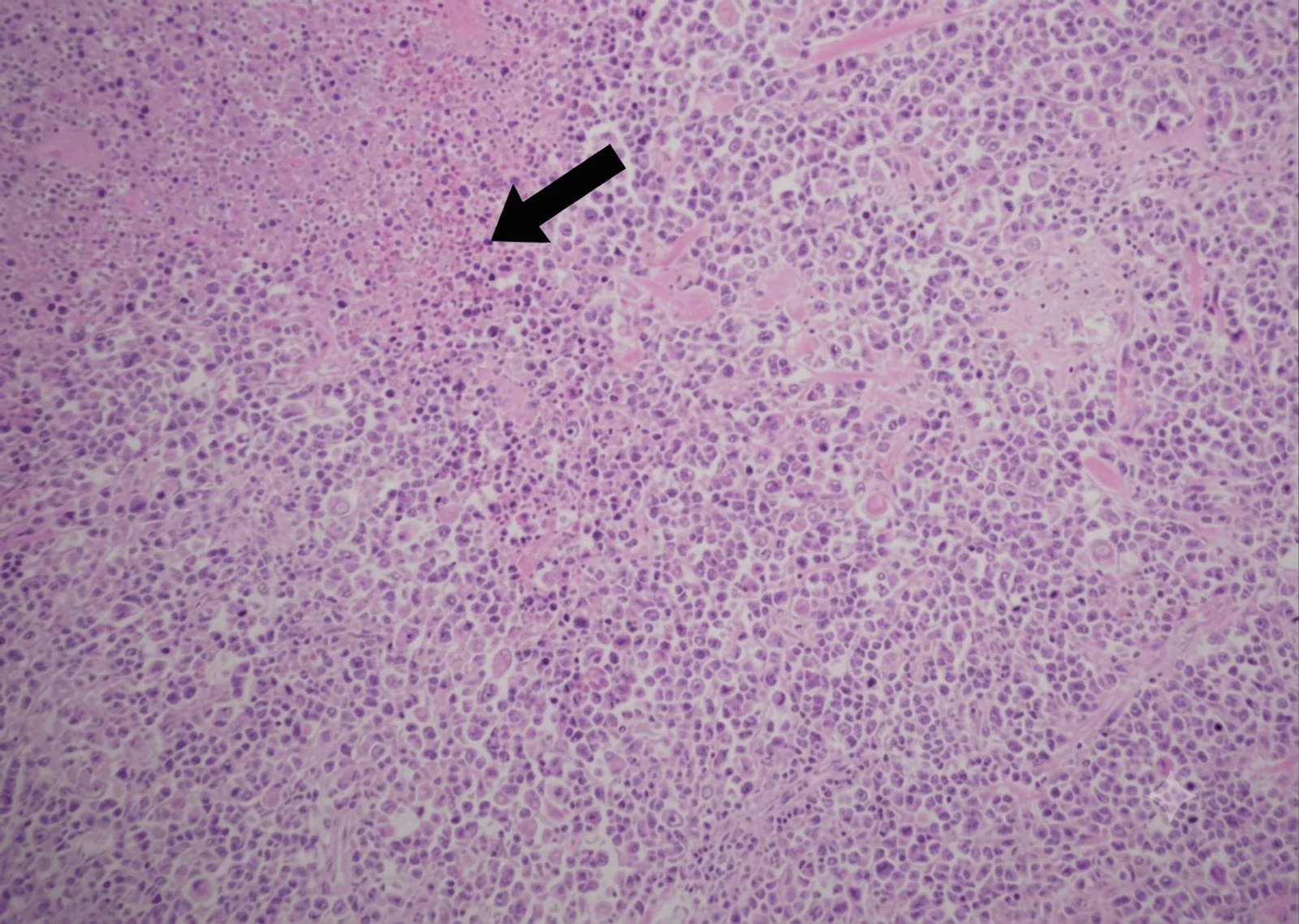
Low-power view of the tumor showing areas of necrosis (arrow).

**Figure 3:**
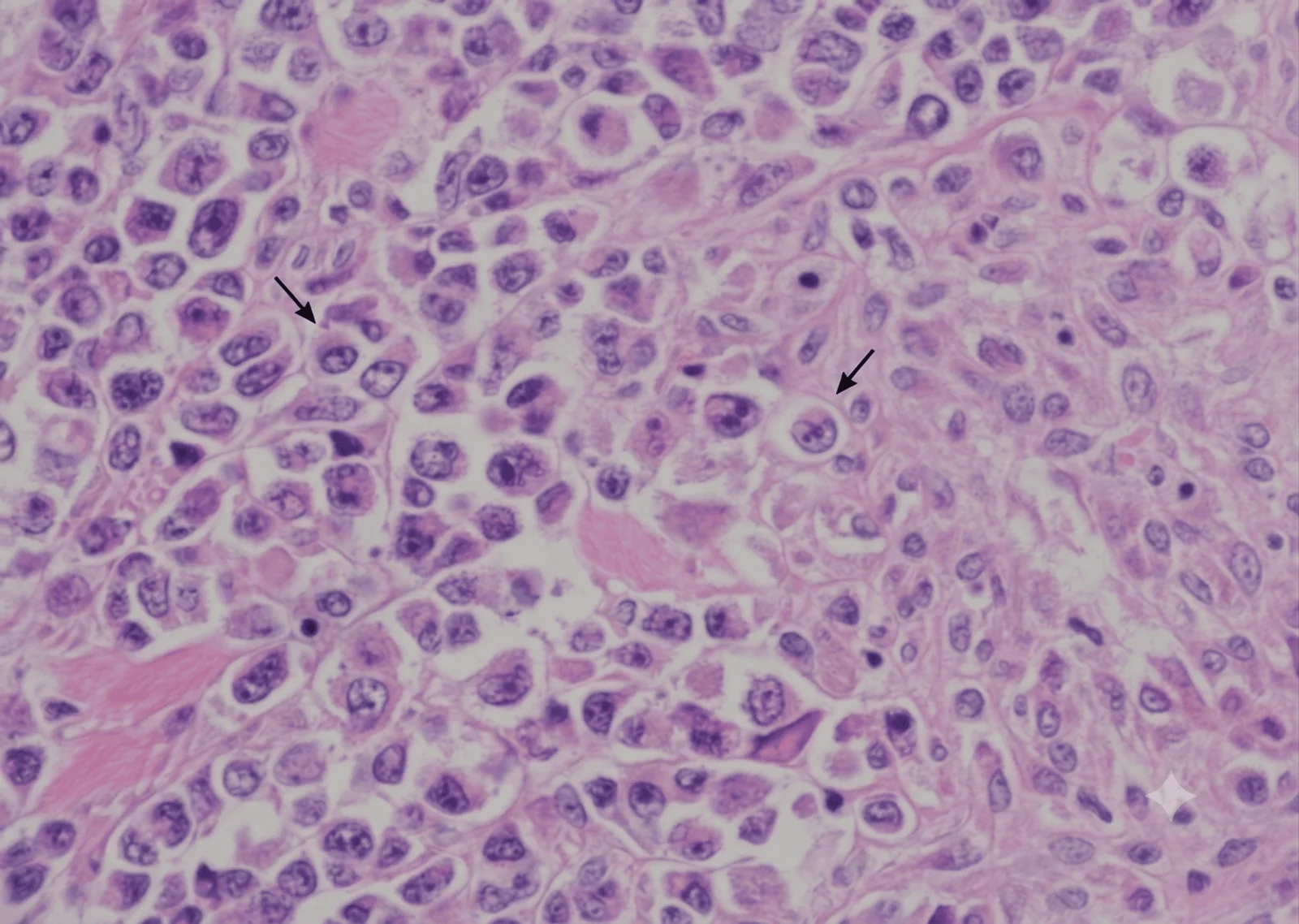
High-power view of the tumor showing highly pleomorphic tumor cells with focal rhabdoid appearance of the malignant cells (arrows).

Histopathological examination confirmed an R0 resection of an almost completely obstructing tumor that had invaded through the bowel wall but had not penetrated the serosa. Microscopically, the tumor demonstrated areas of necrosis, and the morphology was consistent with the previous biopsy findings, confirming SMARCA4-deficient undifferentiated carcinoma (Figures 2, 3). Eighteen of the 33 resected lymph nodes were positive for metastatic disease. The final pathological stage was pT3 pN2b, with extramural vascular invasion and lymphatic invasion.

The patient was referred to the oncology team, and molecular testing demonstrated proficiency for dihydropyrimidine dehydrogenase (DPYD) and mismatch repair (MMR). Tumor profiling identified a KRAS G12D mutation with BRAF wild-type status. At the patient's request, circulating tumor DNA (ctDNA) analysis was performed and was positive (>10%), indicating a high risk of disease recurrence. During this period, she also underwent oocyte retrieval for fertility preservation.

Adjuvant chemotherapy with modified FOLFOX-6 (mFOLFOX-6; 5-fluorouracil, folinic acid, and oxaliplatin) was initiated. Owing to the rarity of this tumor subtype and the absence of established treatment guidelines, management followed standard adjuvant treatment protocols for colorectal adenocarcinoma.

Five days after receiving the first cycle of mFOLFOX-6 in September, the patient was admitted to her local hospital with palpitations, dizziness, and shortness of breath. She was diagnosed with treatment-related heart failure with reduced ejection fraction (35%), which was attributed to 5-fluorouracil cardiotoxicity. During her admission, she developed a sudden fall in hemoglobin, accompanied by severe acute abdominal pain, hypotension, and tachycardia. Imaging demonstrated free pelvic fluid and a large adnexal mass, prompting urgent CT of the thorax, abdomen, and pelvis. This revealed disease recurrence with extensive peritoneal metastases and ascites. MRI, performed on the same day, demonstrated a large lobulated solid pelvic mass measuring 7.8 × 9.1 × 7.6 cm.

She was discharged one week later after agreeing to proceed with a second cycle of FOLFOXIRI (5-fluorouracil, folinic acid, irinotecan, and oxaliplatin). Bevacizumab was considered but was deemed inappropriate because of the high risk of bowel-related complications. However, before further systemic treatment could be administered, she was readmitted with severe abdominal pain and a markedly elevated C-reactive protein concentration of 337 mg/L. Repeat CT of the abdomen and pelvis demonstrated an acute mid-small bowel obstruction, most likely secondary to metastatic infiltration, which was managed conservatively.

Following discussions regarding further systemic anticancer therapy versus best supportive care, the patient elected to receive supportive care alone, as she wished to return home for end-of-life care with the support of the palliative care team. Despite supportive management, her disease progressed rapidly, and she died one month after receiving her first cycle of systemic anticancer therapy.

## Discussion

The rapid progression of this patient's disease is consistent with the aggressive nature of SMARCA4-deficient tumors. Although only one month elapsed between the initial and repeat endoscopies, the tumor demonstrated marked progression and, by the time of surgery, had become almost completely obstructing. Although there was no macroscopic evidence of peritoneal disease at the time of surgery, widespread peritoneal metastases developed rapidly, leading to bowel obstruction and intestinal failure within two months.

The tumor was detected during routine surveillance colonoscopy performed in accordance with the British Society of Gastroenterology guidelines, which recommend surveillance every 1-3 years for patients with extensive or left-sided ulcerative colitis, beginning 8-10 years after diagnosis. The patient had no additional risk factors, uncontrolled disease activity, or family history that would have warranted more frequent endoscopic surveillance. It is likely that, had the diagnosis been delayed until the onset of symptoms, the disease would have progressed even further before detection. There is currently no evidence that vedolizumab is associated with an increased risk of malignancy [9]. Beyond conventional colorectal cancer treatment regimens, several emerging therapeutic strategies are being investigated for SWI/SNF-deficient malignancies, including SMARCA4-deficient tumors. Enhancer of zeste homolog 2 (EZH2) inhibitors have shown promising preclinical activity through synthetic lethality in SMARCA4-deficient models, although clinical evidence remains limited. Immune checkpoint inhibitors have demonstrated variable efficacy across SWI/SNF-deficient cancers, with gastrointestinal SMARCA4-dUT generally exhibiting poorer responses than thoracic primaries. Several early-phase clinical trials are evaluating targeted therapies and immunotherapeutic combinations for SWI/SNF complex-deficient tumors; however, no standardized treatment pathway currently exists for colonic SMARCA4-dUT [10].

Although there was a delay in establishing the tissue diagnosis, during which time the tumor progressed, it remains unclear whether earlier specialist pathological review of the initial biopsy specimens would have altered the clinical outcome, given the highly aggressive nature of SMARCA4-dUT. The diagnostic challenges associated with this tumor subtype are well recognized and largely reflect its unusual morphological features [4]. Given the poor prognosis associated with SMARCA4-dUT and the rapid progression observed in this patient, earlier histological confirmation would likely not have changed the overall clinical course.

Given the rarity of SMARCA4 deficiency in colonic tumors, it is only possible to speculate whether the patient's underlying ulcerative colitis contributed to tumor development. The most common genetic alterations in colitis-associated colorectal cancer involve the Wnt signaling pathway and tumor suppressor genes. However, the role of SMARCA4 (BRG1) in intestinal inflammation and its reduced expression in inflammatory bowel disease provide a plausible biological link between ulcerative colitis and the development of this rare tumor subtype.

The incidence of early-onset colorectal cancer in adults younger than 50 years continues to increase in many Western countries, while the incidence among older adults has stabilized or declined [11]. Younger patients are also more likely to present with advanced-stage disease [12]. The diagnosis of colorectal cancer in this population presents several challenges, including delayed recognition of symptoms because of the patient's young age and the limited evidence supporting the use of fecal immunochemical testing (FIT) in younger individuals [13]. Although dietary and environmental factors undoubtedly contribute to the rising incidence of early-onset colorectal cancer, further research is needed to clarify the role of specific genetic alterations, particularly in patients with a family history of colorectal cancer or those presenting with rapidly progressive disease.

## Conclusions

SMARCA4-dUT are rare and highly aggressive neoplasms associated with a poor prognosis. Colonic SMARCA4-dUT are exceptionally rare, and evidence to guide their management remains limited. Consequently, standardized treatment strategies have yet to be established, highlighting the need for further research to inform surgical and oncological management.

As the incidence of early-onset colorectal cancer continues to increase, a better understanding of the genetic landscape of these tumors will be essential for developing more effective and individualized treatment strategies, particularly for aggressive and advanced disease. Although emerging immunotherapeutic approaches may offer future therapeutic potential, the rarity of tumor subtypes such as SMARCA4-dUT continues to limit opportunities for clinical research and prospective trials.
